# Detection and sequencing of rotavirus among sudanese children

**DOI:** 10.11604/pamj.2017.28.87.11008

**Published:** 2017-09-27

**Authors:** Magzoub Abbas Magzoub, Naser Eldin Bilal, Jalal Ali Bilal, Mohammad Abdulrahman Alzohairy, Bahaeldin Khalid Elamin, Gasim Ibrahim Gasim

**Affiliations:** 1 National Public Health Laboratory, Ministry of Health, Khartoum, Sudan; 2 Faculty of Medical Laboratory Sciences, Khartoum University, Khartoum, Sudan; 3 College of Applied Medical Science, Qassim University, Buraydah, Saudi Arabia; 4 College of Medicine, Qassim University, Qassim, Saudi Arabia; 5 College of Medicine, Bisha University, Bisha, Saudi Arabia

**Keywords:** Rotavirus, VP4, VP7, children, gastroenteritis, Sudan

## Abstract

**Introduction:**

Diarrheal diseases are a big public health problem worldwide, particularly among developing countries. The current study was conducted to detect and characterize group A rotavirus among admitted children with gastroenteritis to the pediatric hospitals, Sudan.

**Methods:**

A total of 755 stool samples were collected from Sudanese children with less than 5 years of age presenting with acute gastroenteritis during the period from April to September 2010. Enzyme-linked immunosorbent assay (ELISA) was used to Detection of Rotavirus antigens. Ribonucleic acid (RNAs) were extracted from rotavirus-positive stool samples using (QIAamp^®^ Viral RNA Mini Kit). (Omniscript^®^ Reverse Transcription kit) was used to convert RNA to complementary Deoxyribonucleic acid (cDNA). The cDNAs were used as template for detection of VP4-P (P for Protease-sensitive) and VP7-G (G for Glycoprotein) genotyping of Rotavirus using nested PCR and sequencing.

**Results:**

Out of the 755 stool samples from children with acute gastroenteritis, 121 were positive for rotavirus A. Among 24 samples that were sequenced; the VP7 predominant G type was G1 (83.3%), followed by G9 (16.7%). Out of these samples, only one VP4 P[8] genotype was detected.

**Conclusion:**

As a conclusion the VP7 predominant G type was G1, followed by G9 whereas only one VP4 genotype was detected and showed similarity to P[8] GenBank strain. It appears that the recently approved rotavirus vaccines in Sudan are well matched to the rotavirus genotypes identified in this study, though more studies are needed.

## Introduction

Among children under five years old, rotavirus A is the most frequent cause of acute gastroenteritis in developing countries [1]. In Sudan rotavirus was the second causes of diarrhea among children [2]. Worldwide there is about 527,000 (range, 475,000-580,000) children die of rotavirus each year this translate into more than 1440 death daily, by other mean, by 5 years of age about 1 of 237 born children would die of rotavirus yearly [3]. There are 11 double-stranded RNA segments of the rotavirus genome that code for viral proteins (VPs) [4]. The outer capsid layer of rotavirus consists of two structural proteins and they were VP4 and VP7. Based on the VP7 and VP4 gene sequences rotaviruses are classified into the (G) and (P) respectively. At present 27 G genotypes (G1-G27) and 35 P genotypes (P1-P35) genotypes have been discriminated so far [5]. Assessment of the potency of rotavirus vaccine against constantly changing strains will depend on continuous global strain surveillance [6]. The diversity of VP4 and VP7 rotavirus strains is a major challenge to the efficacy of the currently used vaccines [7]. Characterization of the common strains in Sudan will have an impact on the implementation of rotavirus vaccines in the country. There is scanty data about the genotypes of rotavirus in Sudan despite its potential impact in the implementation of vaccines. However, the monovalent rotavirus vaccine (Rotarix^®^ GSK Biologicals, Rixensart) was introduced into the most populous state (Khartoum) in Sudan on July 2011 [8]. Therefore, in this study the VP4 and VP7 were selected for sequencing because of their impact upon vaccine implementation. This study was conducted to genetically characterize the VP4 and VP7 rotavirus strains among some Sudanese children with acute gastroenteritis.

## Methods

It is a cross sectional hospital based study conducted in different hospitals from different states of Sudan during period from April to September 2010. A total of 755 stool specimens were collected from children (430 males and 325 females), those children were suffering from acute gastroenteritis and they under 5 years of age. A single stool sample was taken from each child on the first day of hospitalization. After obtaining written informed consent from the parent or guardians stool samples were collected along with demographic data and medical history, using a questionnaire. Ethical approval was obtained from the university of Khartoum ethical and scientific committee. Each sample was collected in a dry clean container and immediately placed in an ice chest and then sent directly to the virology department, National Public Health Laboratory, Khartoum, Sudan for preservation in -80°C. The stool specimens were brought to room temperature. A sum of 100 g from each specimen was diluted in 1 ml of specimen diluents in a clean dry tube. VP6 monoclonal antibody (ELISA) was utilized to detect rotavirus antigen as per the manufacturer's instructions (GA Generic Assay GmbH ^®^, Germany). The Cut-off point was calculated depending on the optical density of the mean negative control plus 0.2 as a constant. Samples reflecting optical densities of more than the predetermined cut-off point were considered positive while those reflecting optical densities equal or less than the set cut-off point were taken as negative. The ELISA positive stool specimens were preserved as raw specimens in ice (- 80°C). The preserved positive stool specimens were used later for RNA extraction according to the manufacturer's instructions of QIAamp^®^ Viral RNA Mini Kit (Qiagen) and the extracted RNA was stored directly at -70°C. To confirm the ELISA results the extracted RNAs were examined by using denaturing formaldehyde agarose gel electrophoresis as described by Magzoub et al [9]. The remaining of the extracted RNAs were stored directly at -70°C and later was used for the reverse transcriptase polymerase chain reaction (RT-PCR). As per the manufacturer's instructions the RNAs were reverse transcribed using the Ominscript Reverse Transcriptase kit (Qiagen) with the use of Oligo-dt primers. The cDNAs products were stored at -20°C

**Figure 1:**
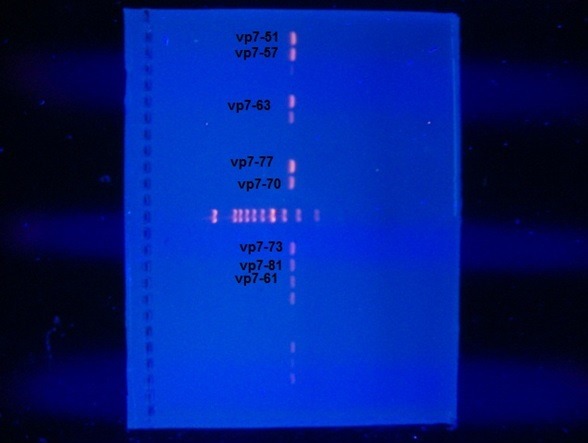
Although it is hospital based study but it seems to be the first study in Sudan in term of rotavirus sequences

**Figure 2:**
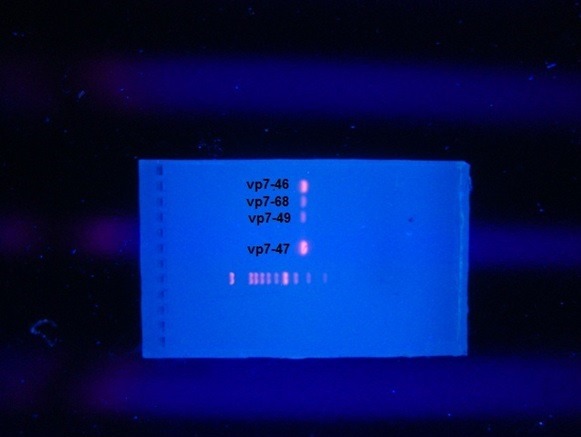
It adds background and an idea of the circulated rotavirus strain in Sudan so, it will help to make a decision of distribute the vaccine in Sudan

For polymerase chain reaction (PCR), initial first round amplification with widely known used primers F con3 (TGG CTT CGC TCA TTT ATA GAC A) and R con2 (ATT TCG GAC CAT TTA TAA CC) for VP7 while the primers F VP7 (TAG TGG ATG TCG TTG ATG G) and R end9 (GGT CAC ATC ATA CAA TTC TAA TCT AAG) were used for VP4 and then a second round of nested PCR amplification was done by the same primers (4). Master mix was prepared, for each reaction 18μl as following: 10μl of HotStarTaq plus Master Mix, 1μl from each forward and reverse primers (F-con3, R-con2= 0.1-0.5 μM) specific for VP4 and (F-VP7, R end9 = 0.1-0.5 μM) primers specific for VP7, 2μl of 10x Concentrated Coral Load and 4μl RNase-Free Water then the master mix was distributed into each PCR tube. Lastly 2μl of the cDNA was added to each reaction. The previous prepared PCR tubes were placed into the wells of the thermal cycler machine (Eppendorf). The program was started with an initial heat activation step at 95°C for 5 minutes followed by 30 cycles, each of denaturation at 94°C for 45seconds, annealing at (48 -50°C) for 45seconds and 1-minute extension at 72°C. Lastly 10 minutes at 72°C then hold at 4°C. After amplification was finished samples were stored overnight at -20°C. Second round PCR amplification conducted by the same previous PCR steps in the first round as nested PCR except 2μl of the PCR product was used instead of cDNA Electrophoresis of PCR product was carried out in a horizontal gel apparatus using 1.8 % (w/v) agarose gel prepared and submerged in Trisacetate- EDTA buffer containing 0.5μg/mL ethidium bromide. 10 μL each PCR product were electrophoresed at 90V for 80 minutes. A 1500 bp DNA marker was used to allow size determination of each PCR product. The specific bands were visualized under UV trans-illuminator and gel was photographed using separated camera. Figure 1 and Figure 2 . The obtained PCR product were shipped to the First Base laboratories, Malaysia for sequencing. The sequence reaction of the product was conducted using ABI Prism BigDye^®^ kits (applied bio system). The nucleotide sequence data were listed by the Sequence Scanner Software. The nucleotide list data were aligned using the Basic Local Alignment Search Tool (BLAST) with Genbank strains. Phylogenetic and molecular evolutionary analysis was carried out using MEGA 5 [10]. The nucleotide sequences obtained in this study were deposited in GenBank. The obtained data was entered in the Statistical Package for the Social Sciences software (SPSS version16). Chi-squire test was used to test for significant differences between the variables and a p value of less than 0.05 was considered as significant.

## Results

Of the 755 fecal samples analyzed, 16% tested positive for rotavirus by ELISA and confirmed by PCR and the specific bands were visualized under UV trans-illuminator and gel was photographed Figure 1 and Figure 2. Of the 121 positive rotavirus children there were (65.3%) males and (34.7%) were females and their age ranged 1-60 month with a mean of 15.6±13.3 months. There is statistically significant difference between gender of rotavirus infected children (P<0.05). Of the 121 rotavirus strains amplifiable by RT-PCR, 24 was able to be sequenced. A total of 24 rotavirus-positive samples were genotyped for G and P. Overall, during the study for VP7 there is 83.3% of strains identified were genotype G1 and 16.7% were G9 while the only rotavirus strain detected was characterized as P[8]. The overall rotavirus genotypic strains were detected among children whom were younger than 2 years of age, of whom (90%) were of VP7 type G1 and (75%) were of type G9 found in children with less than one year of age. There was no significant association between gender and rotavirus strains (p>0.05). The nucleotide sequences obtained in this study were deposited in GenBank under accession numbers [KC741477- KC741478- KC741479- KC741480- KC741481- KC741482- KC741483- KC741484- KC741485- KC741486- KC741487- KC741488- KC741489- KC741490- KC741491- KC741492- KC741493- KC741494- KC741495- KC741496- KC741497- KC741498- KC741499- KC741500].

## Discussion

This study is the a cross sectional hospital based study of rotavirus genotypes in Sudan, and the results support the observation that the G1 and G9 genotype, particularly P[8]G1 and P[8]G9, is the more genotypes spreading throughout Africa [11]. This study described the rotavirus infection among patients presenting to pediatric hospitals in the period from April through September 2010. One of the main aims of this study was to identify and sequencing of the VP7 and VP4 gene segments of the Sudanese For VP4 Only P[8] was detected and showing as example high similarly to wt/PHI/TGE12-045 strain deposit in GenBank accession number KP007144. This finding is similar to a report by Inairo GI et al [12]. Moreover in agreement with our result, recent literature worldwide showed that P[8] is the most prevalent genotype [13, 14]. In this study, the detection rate of 83% VP7 genotype G1 in Sudan is relatively similar to the 82.4% from Egypt [15]. Furthermore, results from eight countries in the Middle East and North Africa showed the prevalence of VP7 genotype G1 to be more than rotavirus strains. 56% [14]. The VP7 G9 genotype has been documented since the early 1980s [16]. Throughout the 1980s and 1990s, G9 was considered to be a very rare strains then beginning to increase as a cause of gastroenteritis and later emerged as the most important strains in developed and developing countries [17]. In Africa the serotype G9 strains have been detected in various countries and sporadically were isolated in South Africa, Botswana, Kenya, and Cameroon and appeared to be circulating at population, however it appears to be the predominant serotype in some settings [18]. In the United States, the G9 genotype was detected through an outbreak in 1995-1996 [19]. Nevertheless, this may not reflect the current prevalence of the G9 genotype, since regional epidemiological reports have shown prevalence as high as 50-90% in some settings [20, 21]. In the present study the 17% rate of G9 is similar to an Iranian [22] and Brazil rate [23]. Moreover, this rate is similar to report from Cameron [24]. On the other hand, our G9 prevalence rate is a little bit higher than the Kuwaiti 10.2% rate [25] and Iraq [26].

In this study the presence of G1P[8] as predominant strains is in agreement of introducing of the monovalent Rotarix^™^ vaccine in Sudan. Moreover the G9 detected strain in the present study had a P[8] antigen which is included in the monovalent Rotarix^™^ vaccine, so severe gastroenteritis due to G1P[8] and G9P[8] strain in Sudan is otherwise expected to wane by implementing the current vaccine. As referred to other publication by Magzoub et al [9], a total of 755 fecal specimens were tested for rotavirus and 16% were positive, a finding similar to Elhag et al [27] and Parashar et al [3] moreover are slightly less than the previous findings in Marocco [28]. In comparison to the rotavirus infection rate between males and females, the study results showed a prevalence rate of rotavirus infections in males higher than females [9] this is in agreement to some worldwide studies which indicated that males are more susceptible to rotavirus infection and actually exhibited a higher rate of rotavirus in their faeces than females [29, 30]. It has to be mentioned that this study dealt with strains isolated from hospitalized patients. Conducting community based surveillance can produce more accurate results in terms of sequencing and characterization of rotavirus strains in order to reflect the true prevalence and circulating genotypes in Sudan.

## Conclusion

As a conclusion from the sequence of VP7 and VP4 it appears that the recently approved rotavirus vaccines in Sudan are well matched to the rotavirus genotypes identified in this study.

### What is known about this topic

- Among children under five years old, rotavirus A is the most frequent cause of acute gastroenteritis in developing countries and Sudan;
- The diversity of VP4 and VP7 rotavirus strains is a major challenge to the efficacy of the currently used vaccines;
- The monovalent rotavirus vaccine (Rotarix^®^ GSK Biologicals, Rixensart) was introduced into the most populous state (Khartoum) in Sudan on July 2011.

### What this study adds

- Although it is hospital based study but it seems to be the first study in Sudan in term of rotavirus sequences;
- It adds background and an idea of the circulated rotavirus strain in Sudan so, it will help to make a decision of distribute the vaccine in Sudan.

## Competing interests

The authors declare no competing interests.
